# Efficient generation of patient-matched malignant and normal primary cell cultures from clear cell renal cell carcinoma patients: clinically relevant models for research and personalized medicine

**DOI:** 10.1186/s12885-016-2539-z

**Published:** 2016-07-16

**Authors:** Nazleen C. Lobo, Craig Gedye, Anthony J. Apostoli, Kevin R. Brown, Joshua Paterson, Natalie Stickle, Michael Robinette, Neil Fleshner, Robert J. Hamilton, Girish Kulkarni, Alexandre Zlotta, Andrew Evans, Antonio Finelli, Jason Moffat, Michael A. S. Jewett, Laurie Ailles

**Affiliations:** Department of Medical Biophysics, University of Toronto, Toronto, ON Canada; School of Biomedical Sciences and Pharmacy, University of Newcastle, Hunter Medical Research Institute, Newcastle, NSW Australia; Princess Margaret Cancer Centre, University Health Network, Toronto, ON Canada; Donnelly Centre and Banting & Best Department of Medical Research, University of Toronto, Toronto, ON Canada; Laboratory Medicine Program, Department of Pathology, University Health Network, Toronto, ON Canada

**Keywords:** Clear cell renal cell carcinoma, In vitro models, Primary cell culture, Renal cancer, *VHL*

## Abstract

**Background:**

Patients with clear cell renal cell carcinoma (ccRCC) have few therapeutic options, as ccRCC is unresponsive to chemotherapy and is highly resistant to radiation. Recently targeted therapies have extended progression-free survival, but responses are variable and no significant overall survival benefit has been achieved. Commercial ccRCC cell lines are often used as model systems to develop novel therapeutic approaches, but these do not accurately recapitulate primary ccRCC tumors at the genomic and transcriptional levels. Furthermore, ccRCC exhibits significant intertumor genetic heterogeneity, and the limited cell lines available fail to represent this aspect of ccRCC. Our objective was to generate accurate preclinical in vitro models of ccRCC using tumor tissues from ccRCC patients.

**Methods:**

ccRCC primary single cell suspensions were cultured in fetal bovine serum (FBS)-containing media or defined serum-free media. Established cultures were characterized by genomic verification of mutations present in the primary tumors, expression of renal epithelial markers, and transcriptional profiling.

**Results:**

The apparent efficiency of primary cell culture establishment was high in both culture conditions, but genotyping revealed that the majority of cultures contained normal, not cancer cells. ccRCC characteristically shows biallelic loss of the von Hippel Lindau (*VHL*) gene, leading to accumulation of hypoxia-inducible factor (HIF) and expression of HIF target genes. Purification of cells based on expression of carbonic anhydrase IX (CA9), a cell surface HIF target, followed by culture in FBS enabled establishment of ccRCC cell cultures with an efficiency of >80 %. Culture in serum-free conditions selected for growth of normal renal proximal tubule epithelial cells. Transcriptional profiling of ccRCC and matched normal cell cultures identified up- and down-regulated networks in ccRCC and comparison to The Cancer Genome Atlas confirmed the clinical validity of our cell cultures.

**Conclusions:**

The ability to establish primary cultures of ccRCC cells and matched normal kidney epithelial cells from almost every patient provides a resource for future development of novel therapies and personalized medicine for ccRCC patients.

**Electronic supplementary material:**

The online version of this article (doi:10.1186/s12885-016-2539-z) contains supplementary material, which is available to authorized users.

## Background

Renal cell carcinoma (RCC) is dominated by the clear cell subtype (ccRCC), which makes up 70 % of all RCC and has a poor prognosis [1]. Biallelic loss or inactivation of the von Hippel Lindau (*VHL*) gene via mutation, deletion or promoter methylation occurs in up to 91 % of sporadic ccRCC [2] and drives a strong pro-survival and angiogenic program due to downstream hypoxia-inducible factor (HIF) accumulation [3].

Cancer cell lines are used extensively to characterize novel anti-cancer therapeutics, but such studies have translated poorly from preclinical studies into clinically useful drugs. Genome-wide analysis shows that ccRCC cell lines have many more copy number alterations than primary ccRCC tissues [4], and transcriptional profiles of cell lines cluster separately from primary tumors [5]. In addition, ccRCC exhibits significant intertumor genetic heterogeneity [6, 7] and the limited cell lines available fail to represent this aspect of ccRCC. A lack of patient-matched normal cells further limits research due to the consequent lack of appropriate controls for experiments and drug screening efforts.

Recently researchers have developed methods for generation of primary tumor-derived cultures such as sphere-forming or adherent cells from brain tumors [8, 9] or 3-D organoids from colorectal cancers [10]. These primary cultures are amenable to high-throughput assays and other manipulations while simultaneously representing more accurate preclinical models of the tumors from which they are derived. We describe a protocol for efficient generation of primary ccRCC and patient-matched normal kidney epithelial cell cultures from ccRCC tissue specimens, providing significant opportunities for development of personalized medicine approaches for ccRCC patients.

## Materials and methods

### Tumor collection and processing

ccRCC samples were obtained from the University Health Network (UHN) and the Cooperative Health Tissue Network from patients providing written consent under UHN Research Ethics Board approval, protocol #09-0828-T (Additional file 1: Table S1). ccRCC tumor tissues were procured within 2–24 h of excision (CHTN samples were shipped overnight from various sites in the United States). Tissue was finely minced using a scalpel, then incubated in a 5 to 10 mL volume of 1× Collagenase/Hyaluronidase and 125 Units/mL DNase (Stem Cell Technologies) with frequent pipetting at 37 °C for two hours. Contaminating red blood cells were lysed with ammonium-chloride/potassium (ACK) lysing buffer (Gibco) and remaining undissociated tissue and cell clumps were filtered out using 70 μm nylon mesh. Dissociated cells were stained with trypan blue, viable cells were counted and cells were either frozen in 90 % fetal bovine serum (FBS)/10 % dimethylsulfoxide or placed into culture.

### Cell culture

Cell suspensions were cultured in Iscove’s Modified Dulbecco’s Medium (IMDM) with 10 % FBS or defined serum-free media (DSFM). DSFM contained DMEM/F12 + Glutamax, 1× B27 supplement (Gibco), 1× non-essential amino acids (Sigma-Aldrich), 1× Lipid Mixture 1 (Sigma-Aldrich), 4 μg/mL Heparin, 1 mM N-acetyl cysteine (Sigma-Aldrich), 10 mM HEPES, 10 ng/mL EGF (Invitrogen), and 10 ng/mL bFGF (Invitrogen). Both culture conditions included 100 Units/mL penicillin, and 100 μg/mL streptomycin. 786–0 and A-498 cells were cultured in IMDM with 10 % FBS, penicillin and streptomycin. All cultured cells were plated at a density of at least 5000 cells/cm^2^ in flasks coated with rat tail collagen type IV (5 μg/cm^2^; BD Biosciences) and incubated at 37 °C, in 5 % CO_2,_ 2 % O_2_. Cell culture media was replaced every three to four days and cultures were passaged at confluence with 0.25 % Trypsin (Wisent) and split between 1:2 and 1:5 depending on growth rate. Cell cultures were monitored for Mycoplasma infection (Mycoalert, Lonza) and cell culture identity verification by short tandem repeat profiling (AmpFLSTR® Identifiler®).

To determine doubling times, primary cell cultures were seeded in collagen-coated 96-well plates in IMDM/10 % FBS at 2500 and 5000 cells/well (6 technical replicates each). 786–0 cells were seeded at a density of 300 cells/well. Plates were incubated in an IncuCyte ZOOM incubator and 4 images/well were taken at each time point. Growth curves based on cell confluence were compiled using IncuCyte ZOOM software from which estimates of doubling times were obtained.

### *VHL* sequencing

DNA was extracted using the Qiagen QIAamp DNA Mini kit. PCR for *VHL* was performed using primer sequences and melting temperatures in Additional file 2: Table S2 and sequenced by Sanger sequencing. Mutations were identified using FinchTV software.

### Flow cytometry

Cells were suspended in Hank’s balanced salt solution with 2 % FBS, blocked with 20 μg/ml mouse IgG on ice for 10 min, then incubated on ice with anti-CD31-PECy7 (1:100; BD Biosciences), anti-CD45-PECy7 (1:100; BD Biosciences) and anti-CA9-PE (Clone 303123, 1:10; R&D Biosystems) for 30 min, washed, and resuspended in Hank’s + 2 % FBS with 1 μg/ml 4′,6-diamidino-2-phenylindole (DAPI). Viable (i.e. DAPI-negative) CD45/CD31-negative cells were sorted into CA9^+^ and CA9^−^ populations using a BD FACSAriaII cell sorter.

### Immunohistochemistry

Adherent cell lines were grown in chamber slides to 50–90 % confluence, washed in PBS, fixed in 4 % paraformaldehyde for 15 min at 4 °C, and subsequently washed and permeabilized in PBS with 0.1 % Tween. Cells were then blocked with 0.5 % BSA, 5 % goat serum and 0.3 % hydrogen peroxide, incubated with primary antibody for 30 min at room temperature, washed, and incubated with a biotinylated goat anti-rabbit or goat anti-mouse secondary antibody, as appropriate, at 1:1000 for 30 min at room temperature. Cells were again washed, incubated with 1:1000 streptavidin-HRP (BD Biosciences) for 30 min at room temperature, washed again, and incubated with 3,3'-diaminobenzidine (DAB) for 5 to 10 min, as directed by the manufacturer (NovaRED Peroxidase Substrate Kit; Vector Laboratories), counterstained with hematoxylin, dehydrated, and coverslipped with histomount. Antibodies and dilutions were as follows: Pan-Cytokeratin, 1:100 (AbCAM); PAX-8, 1:500 (Protein Tech Group); Alkaline Phosphatase, 1:50 (Millipore); Aquaporin1, 1:100 (Abcam); E-Cadherin, 1:100 (Cell Signaling).

### Tumorigenicity in mice

One million *VHL*mut cells were re-suspended in 1:1 PBS/standard growth factor Matrigel (BD Biosciences) and injected under the renal capsule of male NOD/SCID/IL2Rγ^−/−^ mice. 5 mice were injected with each cell line. Animal experimentation followed protocols approved by the University Health Network Animal Care Committee. After 10 weeks, mice were euthanized and tumors were harvested, fixed in formalin, paraffin-embedded and subjected to hematoxylin and eosin staining to assess histology.

### Single Nucleotide Polymorphism (SNP) arrays

Genomic DNA was applied to Illumina Human Omni Express-12 or Omni2.5–8 SNP arrays, hybridized as instructed by the manufacturer, and scanned on the iScan Reader (Illumina). SNP Array data was analyzed with Nexus software using the SNP-FASST2 segmentation algorithm. Probe sets were centered to the median for all samples. Linear systematic correction was applied as follows: The bias values, including percent GC content and fragment length, were used to create a linear model whose parameters were estimated using the least squares method. The estimate was then subtracted from the probe Log2Ratio to obtain the corrected probe values. A minimum difference threshold of 25 % and a *p*-value cutoff of 0.05 were used.

### Transcriptional profiling

mRNA was extracted from snap-frozen tumor tissue and from *VHL*mut and *VHL*wt cultures that were expanded in 10 % FBS conditions at passages 2 to 5. mRNA was isolated using a Qiagen RNeasy Kit and Poly-A enriched mRNA libraries were prepared following the Illumina TruSeq RNA Sample Preparation Kit v2 protocol. Libraries were sized on an Agilent Bioanalyzer, and their concentrations were validated by qPCR. Equimolar amounts of the eighteen different libraries were pooled, and subjected to 51 cycles of single-read sequencing on an Illumina HiSeq 2500 using Illumina V4 chemistry and reagents. The mean number of reads/sample was 40.4 M (min. 34.6 M, max 52.5 M). Reads were aligned to the GRCh37 reference human genome, using Gencode V19 transcript models. Alignment was performed with Tophat (v2.0.13), using default parameters and with the Gencode V19 transcriptome index supplied. The median percentage of aligned reads was 95.8 % (min 91.4 %, max 96.8 %). Gene expression levels were estimated with Cufflinks (v.2.0.2) [11], using default parameters and the Gencode V19 GTF file. All resulting cufflinks output files were merged using a bespoke script written in R (v.3.1.3). For differential expression analysis, a read count matrix was generated with the Bioconductor package ‘*GenomicFeatures*’ (v1.18.7), using the UCSC hg19 “knownGene” transcripts table. Differential expression was determined using *limma* (v3.22.7).

### Gene set enrichment analysis

Three GSEA analyses were performed using the RNAseq data: 1) Using the GSEA v2.2.1 PrerankedTool the *VHL*mut and *VHL*wt signatures (Fig. 5a) were queried against a ranked gene list of TCGA RNAseq data, ranked based on adjusted *p*-values of ccRCC vs. adjacent normal tissue [7]; 2) A gene set database obtained from baderlab.org/GeneSets, which contains 14,082 gene sets from KEGG, MSigDB, Reactome, and GO was queried against a ranked list of the *VHL*mut vs. *VHL*wt RNAseq data, ranked based on adjusted *p*-value; 3) MSigDB Collection C5, consisting of 1454 GO gene sets was queried against the patient tumor vs. *VHL*mut cells ranked gene list (again ranked based on adjusted *p*-value). For the latter two analyses, the outputs from GSEA were imported into Cytoscape v3.2.0 and visualized using the Enrichment Map plugin.

## Results

### Most unselected ccRCC cultures are not cancer cells

CcRCC specimens were processed into primary single cell suspensions, cultured in 10 % FBS or defined serum-free media (DSFM; see Methods) and incubated in 2 % oxygen to improve cell growth and avoid DNA damage [12, 13]. Cells with an epithelial morphology that could be passaged at least 4 times were consistently obtained (56.4 % in DSFM and 71.8 % in FBS; *n* = 39). Eight primary cultures with matched primary tumors and adjacent normal tissues were genotyped using single nucleotide polymorphism (SNP) arrays and, surprisingly, 6 out of 8 cultures established in FBS and *all* cultures in DSFM had a normal genotype (Additional file 10: Figure S1A). Sequencing of *VHL* in primary tumors and cultures verified a patient tumor-matching *VHL* mutation in RCC22 cells grown in FBS (Additional file 10: Figure S1B), while the remaining lines did not recapitulate the patients’ tumor *VHL* mutations.

**Fig. 1:**
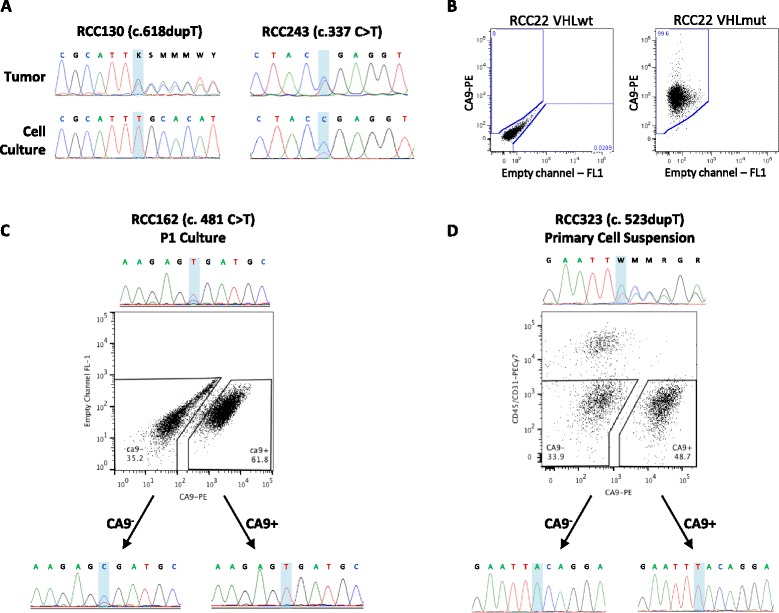
Generation of patient-matched *VHL*mut and *VHL*wt cell cultures from primary ccRCC specimens. **a** RCC130 primary tissue (*left*) contained a mixture of wild-type and mutant cells, and growth in 10 % FBS gave rise to a culture of pure *VHL*mut cells. RCC243 (*right*) contained a mixture of wild-type and mutant cells, and growth in 10 % FBS gave rise to a culture that still contained a mixture wild-type and mutant cells. **b** Flow cytometry profiles of CA9 expression in RCC22 *VHL*wt (*left*) and RCC22 *VHL*mut (*right*). CA9 is expressed on all RCC22 *VHL*mut cells, but not in RCC22 *VHL*wt cells. **c** CA9^+^ and CA9^−^ cells were sorted from RCC162 cells at passage 1 and placed back into culture, and *VHL* sequencing was performed after 2 more passages. CA9^−^ cells continued to give rise to a mixed population of mutant and wild-type cells, whereas CA9^+^ cells gave rise to a culture of pure *VHL*mut cells. **d** CD45/CD31-negative cells were sorted from RCC323 primary tumor tissue single cell suspensions into CA9^+^ and CA9^−^ populations and cultured for 2 passages, then sequenced. CA9^−^ cells gave rise to a culture of pure *VHL*wt cells, and CA9^+^ cells gave rise to a culture of pure *VHL*mut cells

To distinguish cancer vs. normal cells in subsequent experiments, we sequenced the *VHL* gene in a cohort of patients for whom cryopreserved viable single cell suspensions were available. Once patients with sequence-detectable mutations were identified, the cells were thawed and cultured as before. Seven out of seven DSFM cultures were *VHL*-wild-type (*VHL*wt) and only one of seven cases grown in FBS (RCC130) was *VHL*-mutant (*VHL*mut). Another FBS culture, RCC243 contained a mixture of *VHL*mut and *VHL*wt cells at passage two (Fig. 1a).

### Purification of CA9-expressing cells facilitates establishment of ccRCC cultures

*VHL* loss results in HIF accumulation and activation of HIF target genes including carbonic anhydrase IX (CA9), which is constitutively upregulated in *VHL*-mutant cells and acts as a diagnostic biomarker in this disease [14, 15]. Supporting this, RCC22 *VHL*mut cells expressed CA9, while RCC22 *VHL*wt cells did not (Fig. 1b). To determine whether this known ccRCC cell surface marker could be used to select ccRCC cells present in mixtures of cancer and normal cells, we purified CA9^+^ and CA9^−^ cells from cryopreserved primary single cell suspensions or early passage 10 % FBS cultures using fluorescence-activated cell sorting, and (re)cultured them in 10 % FBS (Fig. 1c, d). Cells were passaged twice before re-sequencing the *VHL* gene. The efficiency of *VHL*mut cell culture establishment increased to 37.5 % (6 out of 16 attempts) upon sorting of CA9^+^ cells from early passage cultures, and to 84.6 % (11 out of 13 attempts) upon sorting of CA9^+^ cells from primary cell suspensions (Table 1). The CA9^−^ population gave rise to *VHL*wt cultures 75 % of the time vs. 66.7 % of the time in early passage vs. primary cell suspensions, respectively. We also established pure *VHL*wt cells 70.6 % of the time when primary cell suspensions were plated directly into DSFM without sorting. In 4 cases where *VHL*wt cultures were not established by plating directly in DSFM, they were established from purified CA9^−^ cells. Once established, *VHL*wt cells grew efficiently in both FBS and DSFM conditions, and thus were transitioned to FBS conditions once verified to be *VHL*wt by Sanger sequencing. By contrast, *VHL*mut lines proliferated only in FBS. *VHL*mut and *VHL*wt cells could not be distinguished based on morphology (Additional file 10: Figure S2). Re-sequencing at later passages (up to 20) verified that the *VHL* status of both mutant and wild-type cultures was maintained. Overall, we have successfully established 17 *VHL*mut ccRCC cell cultures, of which 16 have patient-matched *VHL*wt cell cultures (Additional file 3: Table S3).

**Table 1:** Efficiency of *VHL*mut and *VHL*wt primary culture establishment upon isolation of CA9+ and CA9- cells from early passage cultures or primary single cell suspensions

	Cells sorted at P0 to P2			Cells sorted from primary single cell suspension		
Tumor ID	Passage	CA9+	CA9-	CA9+	CA9-	Unsorted (DSFM)
118^c^	0	pure T	50/50^a^	pure T	50/50^a^	no line
137^c^	0	mixture	no line	pure T	pure N	no line
138^c^	0	mixture	no line	pure T	pure N	no line
149^c^		ND	ND	pure T	pure N	pure N
162^c^	0	pure T	50/50^a^	ND	ND	50/50^a^
171^c^	2	mixture	pure N	pure T	pure N	ND
183	1	pure N	pure N	ND	ND	pure N
200		ND	ND	mixture	pure N	no line
222^c^	0	mixture	pure N	pure T	pure N	pure N
243^c^	2	pure T	mixture	ND	ND	pure N
267	0	pure N	pure N	ND	ND	pure N
271^c^	0	pure T	mixture	pure T	mixture	pure N
291^c^	0	pure N	pure N	pure T	no line	ND
294	0	mixture	pure N	no line	ND	pure N
323^c^	0	pure T	pure N	pure T	mixture	pure N
364^b^		ND	ND	pure T	no line	no line
373	0	mixture	pure N	ND	ND	pure N
400^c^	0	pure N	pure N	pure T	pure N	pure N
407^c^	0	pure T	pure N	ND	ND	pure N
		6 of 16 ccRCC cultures (37.5 %)	12 of 16 normal cultures (75 %)	11 of 13 ccRCC cultures (84.6 %)	8 of 12 normal cultures (66.7 %)	12 of 17 normal cultures (70.6 %)

*T* tumor, *N* normal, *ND* not done

^a^Patient had a germline *VHL* mutation, therefore normal cell cultures are heterozygous

^b^ *VHL*mut cell line only was generated, *n* = 1

^c^ *VHL*mut and *VHL*wt pairs were generated, *n* = 13

### *VHL*wt cell cultures are renal proximal tubule epithelial cells

**Fig. 2:**
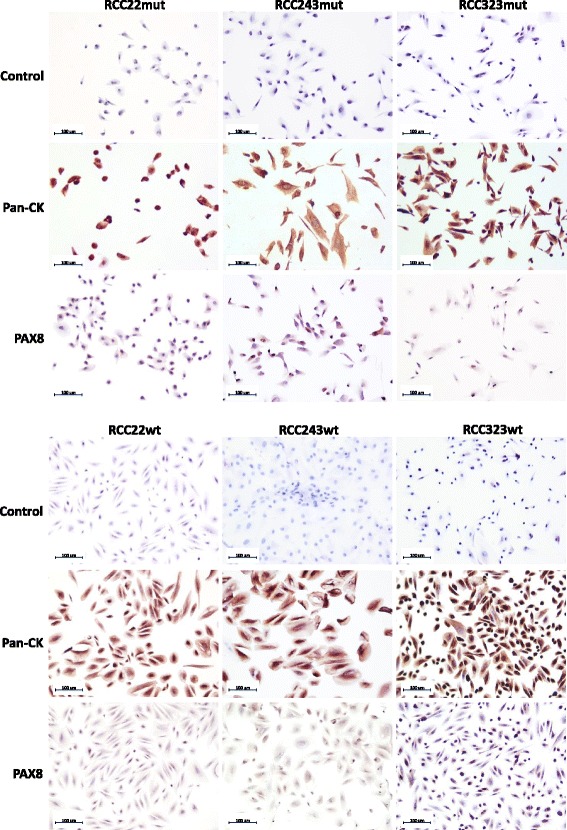
*VHL*mut and *VHL*wt cultures contain renal epithelial cells. *VHL*mut (*top*) and *VHL*wt (*bottom*) cultures stained positively with pan-Cytokeratin (Pan-CK) and PAX8 antibodies, verifying their identity as renal epithelial cells. Three representative *VHL*mut/*VHL*wt pairs are shown. Scale bar = 100 μm

**Fig. 3:**
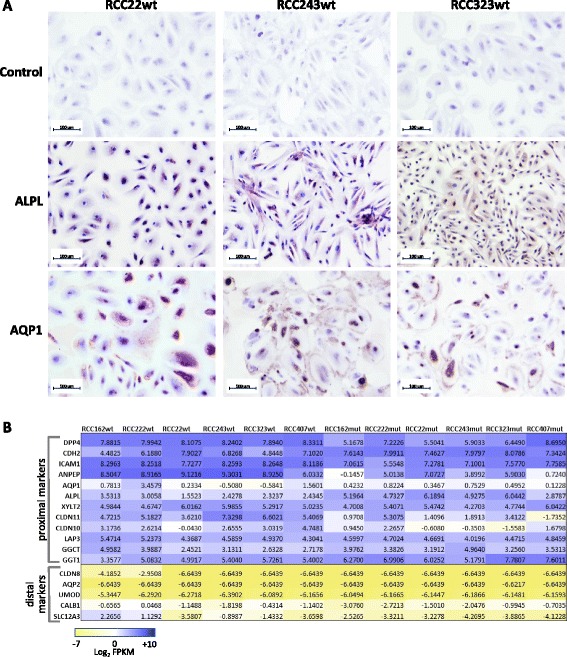
*VHL*wt cultures are renal proximal tubule epithelial cells. **a** *VHL*wt cultures stained positively for proximal tubule markers Alkaline Phosphatase (ALPL) and Aquaporin1 (AQP1). Scale bar = 100 μm. **b** Expression values for proximal and distal tubule markers in *VHL*wt and *VHL*mut cell cultures obtained from RNAseq data, indicating that both *VHL*wt and *VHL*mut cells express proximal but not distal tubule markers

We next performed immunohistochemistry for renal epithelial markers in *VHL*wt and *VHL*mut cultures. All were positive for pan-Cytokeratin and PAX8 (Fig. 2), a transcription factor expressed only in epithelial cells of the adult kidney, in 98 % of ccRCCs [16], and in the thyroid gland and Müllerian duct derived tissues, but not other tissues [17]. These results support the renal epithelial origin of the *VHL*mut and *VHL*wt cultures. *VHL*wt cells are also positive for proximal tubule markers Alkaline Phosphatase [18] and Aquaporin1 [19] (Fig. 3a) and negative for distal tubule markers E-Cadherin and Calbindin1 (Additional file 10: Figure S3). Furthermore, analysis of the expression of proximal and distal tubule markers in RNAseq data generated from 6 pairs of *VHL*mut and *VHL*wt cells indicates consistently higher expression of proximal tubule markers in *VHL*wt cells (Fig. 3b), supporting their identity as proximal tubule epithelial cells. *VHL*mut cells also express proximal tubule markers, as expected [20].

### Growth characteristics of *VHL*mut and *VHL*wt cells

To assess growth kinetics of *VHL*mut and *VHL*wt cultures, growth curves were generated using an Incucyte ZOOM live cell imaging apparatus. The doubling times of 7 *VHL*mut cultures averaged 130 h (range 44 to 219; Additional file 4: Table S4). In contrast to *VHL*mut cultures, the commercial cell line 786–0 had a doubling time of 16 h. These 7 cultures have been passaged from 12 to 20 times and have not yet shown signs of senescence. In contrast, the growth of *VHL*wt cultures slows between passages 6 and 12, though significant expansion and cryopreservation at early passage is possible. To assess tumorigenicity, 1 million *VHL*mut cells were injected under the renal capsule of five NOD/SCID/IL2Rγ^−/−^ mice and 2 out of 4 *VHL*mut cultures consistently generated tumor xenografts with ccRCC histology within 10 weeks (Additional file 10: Figure S4).

### Molecular analysis of *VHL*mut and *VHL*wt primary cultures

**Fig. 4:**
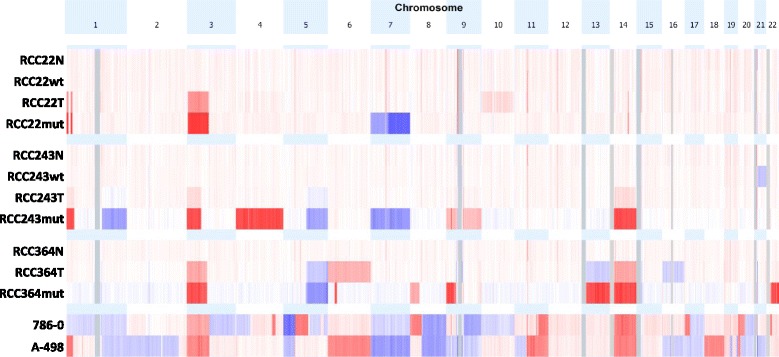
Copy number profiles of *VHL*mut and *VHL*wt cultures and their matched tissues. Three *VHL*mut cultures that had reached passage 20 were profiled using Illumina Human Omni2.5–8 SNP arrays. DNA samples obtained from the matching primary tumor and adjacent normal kidney tissue were profiled in parallel, as well as two commercially available cell lines, 786–0 and A-498. For RCC22 and RCC243, the matched *VHL*wt line was also profiled; RCC364 does not have a matched *VHL*wt line. Amplifications are shown in blue and deletions in red. *VHL*wt cell lines do not display any gross genomic alterations, whereas *VHL*mut cell lines contain alterations matching the tumors from which they were derived. 786–0 and A-498 (*bottom*), have considerably larger numbers of alterations compared to primary tumors and RCC *VHL*mut cultures

To assess genotype stability in vitro, we performed SNP arrays on three *VHL*mut cultures that had reached 20 passages, two matched earlier passage *VHL*wt cultures, plus their matched primary tumors and adjacent normal tissues. Two commercially available ccRCC cell lines, 786–0 and A-498 were also analyzed (Fig. 4). *VHL*wt cell cultures do not exhibit gross genomic abnormalities. *VHL*mut cultures match the copy number alterations (CNAs) of their paired parental tumors, and display a few additional alterations that were not obvious in primary tumors: RCC22mut and RCC243mut have gain of chromosome 7, RCC243mut also has loss of chromosome 4, and RCC364mut has 2 deletions, one in chromosome 8 and one in chromosome 9. In comparison, 786–0 and A-498 had considerably more CNAs than primary ccRCCs.

**Fig. 5:**
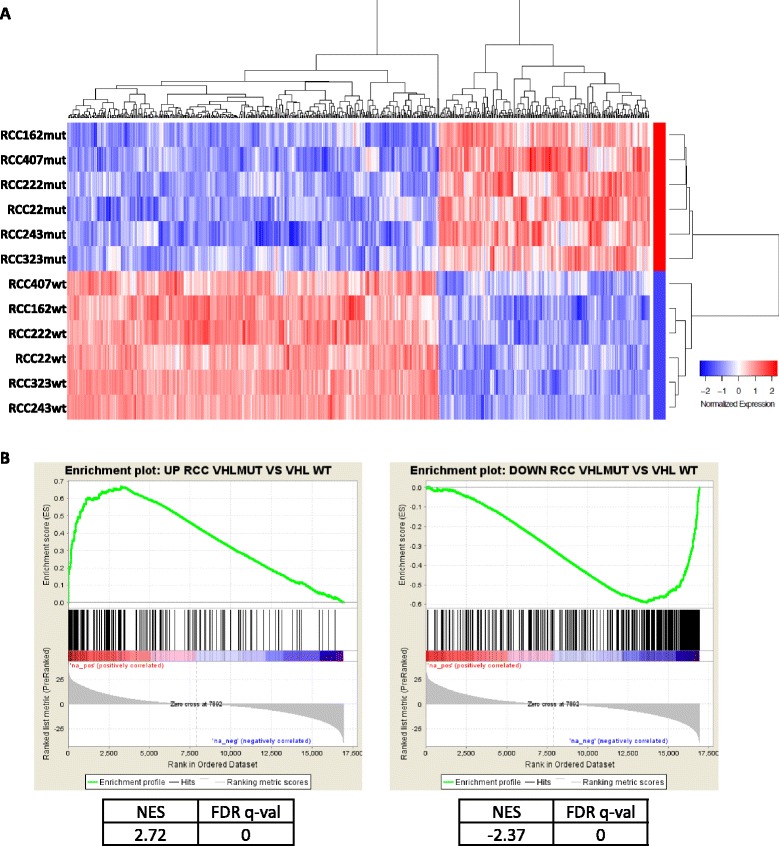
Differentially expressed genes in *VHL*mut vs. *VHL*wt cells. **a** Heatmap showing the 593 differentially expressed protein-coding genes between *VHL*mut and *VHL*wt cells (cut-off of *p* ≤ 0.01 and 2-fold change). **b** Gene set enrichment analysis (GSEA) plots of the 211 *VHL*mut-associated genes (*left*) and 382 *VHL*wt-associated genes (*right*) in the TCGA ccRCC dataset. NES = normalized enrichment score; FDR q-val = false discovery rate q value. A gene set is considered significant with FDR < 0.25

We performed RNAseq on 6 *VHL*mut/*VHL*wt cell culture pairs grown in 10 % FBS at passage 2 to 5, as well as RNA extracted from the matched primary tumor tissues. RNAseq data has been deposited in NCBI’s Gene Expression Omnibus [21] and are accessible through GEO Series accession number GSE74958. Upon unsupervised clustering of gene expression profiles the tumor tissues clustered discretely from the cultured cells, and the *VHL*mut and *VHL*wt cultures formed discrete subclusters, indicating distinct expression profiles within each sample type (Additional file 10: Figure S5). 593 differentially expressed protein-coding genes (cut-off of 2-fold and adjusted *p*-value ≤0.01) were identified between *VHL*mut and *VHL*wt cell cultures (Fig. 5a). Genes differentially expressed in *VHL*mut cells included hypoxia response genes and genes related to metabolism, as expected (Additional file 5: Table S5, Additional file 10: Figure S6). Gene set enrichment analysis (GSEA) of the *VHL*mut and *VHL*wt gene signatures against RNAseq data from patient-matched primary tumor and adjacent normal tissues generated by The Cancer Genome Atlas (TCGA) [7] showed significant enrichment of the *VHL*mut signature in ccRCC tumor tissues, and of the *VHL*wt signature in normal adjacent kidney tissues, respectively (Fig. 5b).

**Fig. 6:**
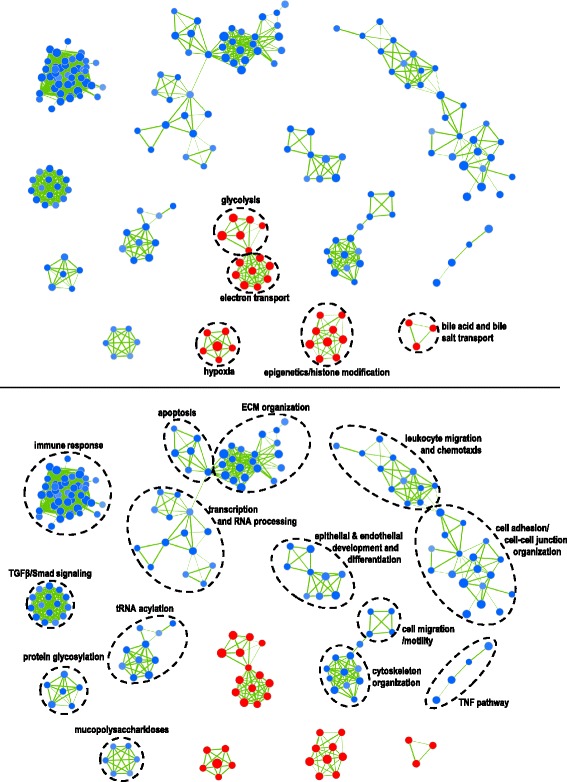
Schematic of workflow to generate matched *VHL*mut and *VHL*wt cultures from surgically resected ccRCC samples. Tumor tissue that has been surgically resected is digested with enzymes to generate a single cell suspension, as described in the Methods. A portion of the tissue is used to extract DNA for *VHL* gene sequencing. The cell suspension can be viably frozen until sequencing results are obtained, if desired. An aliquot of cells is cultured in DSFM to generate a *VHL*wt culture. Remaining cells are stained with antibodies to CD45 and CD31 to allow exclusion of contaminating immune and endothelial cells, and the CA9+ and CA9- fractions are isolated by FACS and plated in media containing FBS. We showed that when both methods for generating a *VHL*wt culture were performed, the success rate increased to 90 % (9 out of 10 attempts; Table 1). After at least 2 passages, DNA is isolated from the cultured cells and sequenced to verify their identity as *VHL*mut cells and *VHL*wt cells. Cultures should be monitored every few passages to ensure identity and *VHL* mutation status

To gain a better understanding of differentially expressed networks in *VHL*mut vs. *VHL*wt cells, we also performed GSEA of a compiled database of gene sets relating to biological processes, molecular functions and pathways [22] against a ranked gene list of our entire expression dataset [23]. Results were visualized using Cytoscape with the Enrichment Map plugin [22]. Only a few networks were up-regulated in *VHL*mut cells, including glycolysis and electron transport, hypoxia/oxygen response, epigenetics/histone modification and bile acid and bile salt transport (Fig. 6, Additional file 6: Table S6). The top differentially expressed genes associated with each of these networks are listed in Additional file 7: Table S7. A larger number of pathways and networks were enriched in *VHL*wt cells, including cell-cell and cell-matrix interactions, focal adhesion and cytoskeleton organization, epithelial and endothelial differentiation, growth factor pathways such as TGFβ and TNF, glycosylation, and RNA processing and transport (Additional file 6: Table S6).

An analysis of differentially expressed genes between *VHL*mut cell cultures and the matched tumors from which they were derived revealed 4312 differentially expressed genes (cut-off 2-fold and adjusted *p*-value ≤0.01; Additional file 8: Table S8). Interestingly, there were many more genes lost in *VHL*mut cells as compared to primary tumors (2781) than gained (1531). As above, GSEA of gene ontology (GO) terms was performed using the ranked gene list of the primary tumor vs. *VHL*mut genes, and the results were again visualized using the Cytoscape Enrichment Map plugin (Additional file 10: Figure S7). Networks that were lost in *VHL*mut cultures consisted almost entirely of networks associated with immune responses, and mitochondrial respiration. The only network found to be gained in the *VHL*mut cultures as compared to the primary tumors was proliferation (Additional file 9: Table S9).

## Discussion

Our finding that unselected cultures derived from primary ccRCC specimens contained predominantly genomically normal epithelial cells was surprising and highlights the importance of genotypic validation of newly established cultures in comparison to the patient tissues from which they are derived. Others have reported that normal cells out-compete malignant cells in primary cancer cultures [10, 24, 25], possibly explaining the poor efficiency of cell line derivation from most solid tumors [26] where lines can often be established, but cannot be maintained. Our protocol establishes accurate *VHL*mut ccRCC cultures that can be passaged at least 20 times. This method will allow researchers to avoid use of extensively passaged lines of uncertain provenance, instead enabling use of early passage, clinically relevant ccRCC cultures for basic studies.

The purification of ccRCC cells expressing CA9 increased both culture accuracy and efficiency substantially for samples bearing *VHL* mutations. This method can be applied to any specimen yielding at least 1 million viable cells upon processing, thus one limitation is the inability to generate cultures from small specimens, such as biopsies. While not all ccRCC tumors have a detectable *VHL* mutation, *VHL* loss due to biallelic deletion or epigenetic silencing occurs in many of these patients [2]. While we have not formally tested CA9 sorting in these patients, our method is likely also applicable in these cases. Indeed, CA9 is expressed in the vast majority (94 %) of ccRCC patients [15].

Our results indicated that cultures established in DSFM were normal renal proximal tubule epithelial cells, the presumed cell of origin for ccRCC [1]. The outgrowth of normal epithelial cells was surprising given that these cells are not obviously present upon microscopic examination of tumor tissues, leading us to conclude that tumor tissues contain rare residual normal cells with a strong growth advantage over ccRCC cells in DSFM. These culture conditions are remarkably similar to conditions established decades ago for the culture of proximal tubule cells from normal kidneys [27]. Interestingly, our results indicate that DSFM culture conditions that support other cancers like glioblastoma multiforme, prostate cancer, and colorectal cancer [28] do not permit ccRCC cell growth; when we transferred *VHL*mut cultures from FBS to DSFM they failed to proliferate, suggesting the intriguing possibility that identification of essential growth factors for ccRCC cells could reveal novel therapeutic targets. Importantly, the differential growth requirements of *VHL*mut ccRCC cells and *VHL*wt normal proximal tubule epithelial cells can be exploited to consistently generate patient-matched normal cultures for virtually every cancer culture, even in the absence of adjacent normal tissue. While these cells senesce at passage 6 to 12, this could potentially be overcome by immortalization with a human telomerase reverse transcriptase (*hTERT*) construct.

Primary *VHL*mut cultures had doubling times ranging from 44 to >200 h, in striking contrast to the 786–0 doubling time of 16 h. This more accurately reflects the heterogeneity and growth kinetics observed in patients and primary xenografts. Cultures were readily established from tumors of varying stages and grades (Additional file 1: Table S1) suggesting no bias for success from more aggressive cancers. Two of four cultures tested to date generate xenografts with characteristic ccRCC clear cell histology. The failure of some cultures to initiate tumors in mice may be due to an insufficient cell dose, or to the eight to ten week incubation period. In our experience patient-derived xenografts initiated directly from primary tissues require four to six months to engraft [29], thus longer incubation times may be required. This would further indicate that our primary cultures are more reflective of patients’ cancers than commercially available cell lines.

SNP array analyses indicated that, as shown by Beroukhim et al. [4], the cell lines 786–0 and A-498 have a large number of CNAs. In our two SNP array experiments we saw a single sample, RCC99 (Additional file 10: Figure S1) that also had substantial CNAs, but this is not typical of ccRCC, as seen in larger-scale genomic studies such as that of Beroukhim et al. [4] and the TCGA [7]. Thus while it is possible that cell lines such as 786–0 and A-498 were derived from such samples, the more likely explanation is that many genomic alterations are acquired during long-term passage in culture. At passage 20 our *VHL*mut cultures contained a small number of alterations that were not apparent in their parent tumors, but they did not have the extreme aneuploidy seen in commercially available ccRCC cell lines [4]. This is likely related to their relatively low passage number, and in part to their maintenance in a low oxygen environment, which has been found to increase plating efficiency and lifespan of cells in culture [12], as well as lead to less DNA damage and fewer stress responses [13]. The novel CNAs that were observed in *VHL*mut cultures compared to their matched primary tumors were concordant with common events in ccRCC such as trisomy 7 [30], suggesting that clones with these alterations may have been present at a subclonal level within the primary tumors, or that cultures were derived from a subclone that was not present in the portion of tumor used for DNA analysis due to regional genetic heterogeneity in ccRCC [31]. Future studies utilizing cultures derived from multiple biopsies per patient may provide valuable insights into biological differences between subclones. Alternatively, these alterations could have been acquired during passage, suggesting that despite our best efforts we did not entirely avoid the acquisition of genomic alterations during culture, and supporting the need to utilize in vitro models at early passage.

Transcriptional profiling identified differentially expressed genes between *VHL*mut and *VHL*wt cultures. Our *VHL*mut gene signature is highly enriched in patient ccRCC samples analyzed by the TCGA and transcriptional networks were identified that would be predicted as a consequence of *VHL* loss: activation of the hypoxic response and increased glycolysis due to HIF stabilization. These results support the use of our *VHL*mut/*VHL*wt paired cultures as representative models of ccRCC biology. The observed increase in genes involved in electron transport/oxidative metabolism, which has been shown to be down regulated in other transcriptional analyses of ccRCC [32] was initially unexpected, however, some of the genes that are present in this network are in fact involved in negative regulation of electron transport; for example, the most significant gene in that network, NDUFA4L2 (see Additional file 7: Table S7) has been shown to be involved in lowering mitochondrial oxygen consumption and Complex I mitochondrial activity, causing a shift from mitochondrial respiration to anaerobic glycolysis [33]. Other genes in the network are associated with other metabolic processes such as adipogenesis and glycogen synthesis, in line with the fat and glycogen storage phenotype that is the hallmark of “clear cell” RCC [34]. In addition, recent findings suggest that as ccRCC cell lines adapt to in vitro culture they derive more of their metabolic demands from oxidative metabolism than directly isolated ex vivo cells [35]. We also noted a network of epigenetics-related nodes, concordant with recent findings that widespread deregulation of chromatin status occurs in ccRCC due to activation of hypoxia-related pathways, as well as mutations in epigenetic regulatory genes [6]. Our observation that many networks are down-regulated, including transcription and RNA processing, is also consistent with observations that hypoxia induces a signature of chromatin modifications and global repression of transcription [36]. Additional pathways repressed in *VHL*mut cells include functions related to normal epithelial biology and tissue homeostasis, such as cytoskeleton organization, cell-cell interactions, extracellular matrix organization and apoptosis, whose loss likely plays a significant role in the malignant behavior of ccRCC.

Comparison of transcriptional profiles between *VHL*mut cells and their matched primary tumor specimens revealed >4000 differentially expressed genes. At first glance this suggests that the *VHL*mut cultures are not representative of the primary tumors from which they were derived at the transcriptional level. However, a deeper analysis of the differentially expressed genes in the tumor tissues revealed that these were related primarily to a wide range of immune functions, suggesting that the expression of these genes was lost due to the presence of large numbers of infiltrating immune cells in the tumor tissues that were not present in the cultures. Likewise, a higher expression of mitochondrial respiration-associated genes in the tumor tissue likely also reflects the presence of stromal cells within the tumor tissue that do not have *VHL* mutations and thus maintain normal levels of mitochondrial metabolism. Indeed, a recent study in which an algorithm was developed to estimate the fraction of stromal and immune cells in tumor samples from gene expression data [37] showed that, upon analysis of gene expression data sets from ten tumor types, ccRCC contained a particularly high level of immune signature expression that correlated with the tumor cellularity as assessed by DNA analysis. The predominant network upregulated in *VHL*mut cultures compared to the matched primary tissues was proliferation, which is also not surprising given that the cultured cells are proliferating in response to culture conditions, whereas the proliferation index in ccRCC tumor tissues as defined by Ki67 staining is in general quite low (median 7.3 % in one study of 176 patients) [38]. Thus overall, while a large number of differentially expressed genes were identified between tumor tissues and *VHL*mut cultures, the majority of these were related to the loss of immune cells in the cultures, and the stimulation of cell proliferation in vitro.

## Conclusions

**Fig. 7:**
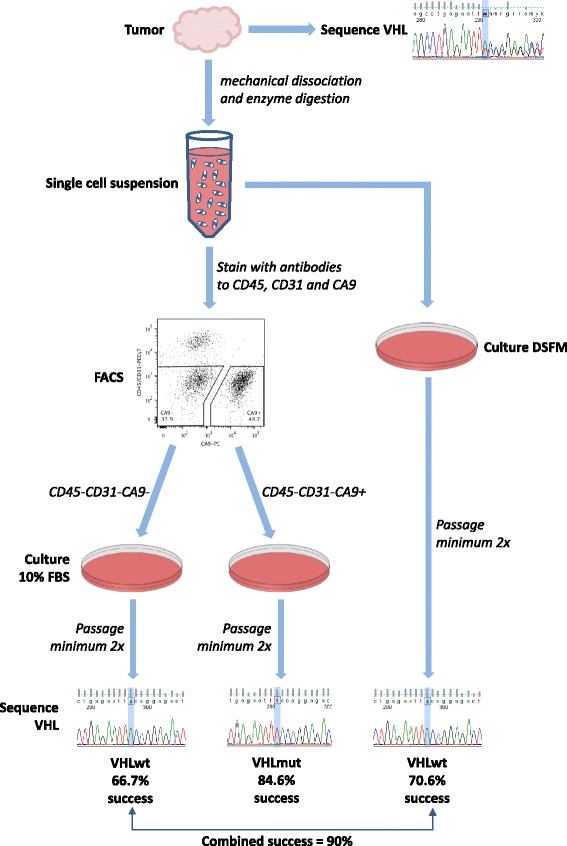
Enrichment map of *VHL*mut vs. *VHL*wt gene expression profiles. To visualize networks and pathways differentially expressed between *VHL*mut and *VHL*wt cell cultures, we performed gene set enrichment analysis, and visualized the results using the Cytoscape Enrichment Map plugin, as described in the Methods. *VHL*mut related networks are shown in red (*top*) and *VHL*wt related networks are shown in blue (*bottom*)

We have exploited the unique biology of ccRCC to develop a protocol for the reproducible and accurate generation of ccRCC and patient-matched normal proximal tubule epithelial cell cultures (Fig. 7). Transcriptional profiles recapitulate those of primary ccRCC tissues and verify the expression of pathways known to be key components of ccRCC cell biology. The high efficiency of cell culture establishment means that large numbers of cultures can be established from individual patients, allowing studies to be performed that take into consideration the genetic diversity of patients. The ability to generate patient-matched normal cultures from every patient provides a powerful tool for exploring molecular and functional differences between normal and cancer cells and provides ideal reagents for screening for cancer-specific compounds. Our protocol thus overcomes the severe limitations imposed by use of commercial cell lines, and provides a unique resource for the development of personalized therapeutic approaches in a disease that is in great need of novel therapies.

## Abbreviations

bFGF, basic fibroblast growth factor; CA9, carbonic anhydrase IX; ccRCC, clear cell renal cell carcinoma; CNA, copy number alteration; DNA, deoxyribonucleic acid; DSFM, defined serum-free media; EGF, epidermal growth factor; FBS, fetal bovine serum; GO, gene ontology; GSEA, gene set enrichment analysis; HIF, hypoxia inducible factor; IMDM, Iscove’s modified Dulbecco’s media; KEGG, Kyoto encyclopedia of genes and genomes; NOD/SCID/IL2Rγ−/−, Nonobese diabetic/severe combined immunodeficient/interleukin 2-receptor gamma knockout; RCC, renal cell carcinoma; RNA, ribonucleic acid; RNAseq, RNA sequencing; SNP, single nucleotide polymorphism; TCGA, the cancer genome atlas; TGFβ, transforming growth factor beta; TNF, tumor necrosis factor; VHL, von Hippel Lindau; VHLmut, von Hippel Lindau mutant; VHLwt, von Hippel Lindau wild-type

## Additional files

Additional file 1: Table S1. Clinical characteristics of samples used to generate cell lines. (XLS 36 kb) Additional file 2: Table S2. Primers for *VHL* sequencing. (XLS 35 kb) Additional file 3: Table S3. Summary of cell cultures established. (XLS 40 kb) Additional file 4: Table S4. Doubling times of *VHL*mut and *VHL*wt ccRCC cultures. (XLS 25 kb) Additional file 5: Table S5. Differentially expressed genes between *VHL*mut and *VHL*wt cell cultures. (XLSX 58 kb) Additional file 6: Table S6. Differrentially expressed networks between *VHL*mut and *VHL*wt cell cultures. (XLS 66 kb) Additional file 7: Table S7. Genes associated with the *VHL*mut networks shown in Fig. 6. (XLSX 14 kb) Additional file 8: Table S8. Differentially expressed genes between matched tumor tissue and *VHL*mut cultures. (XLSX 1.05 mb) Additional file 9: Table S9. Differentially expressed genes between matched tumor tissue and *VHL*mut cultures. (XLSX 16 kb) Additional file 10: Figure S1. Copy number profiles of primary cultures and matched primary tumors and adjacent normal tissues. **Figure S2**. Morphology of *VHL*mut vs. *VHL*wt cultures. **Figure S3**. VHLwt cells do not express distal tubule markers. **Figure S4**. Xenografts derived from RCC#243mut and RCC#407mut cultures. **Figure S5**. Hierarchical clustering heatmap of Pearson correlation coefficients between matched *VHL*mut cells, *VHL*wt cells and primary tumor tissues. **Figure S6**. Functional annotation of 211 *VHL*mut-associated genes (red) and 382 *VHL*wt-associated genes. **Figure S7**. Enrichment map of tumor tissue vs. *VHL*mut culture gene expression profiles. (PPTX 10688 kb)
