# Supplementary material for: Efficient generation of patient-matched malignant and normal primary cell cultures from clear cell renal cell carcinoma patients: clinically relevant models for research and personalized medicine
Source: BMC Cancer. 2016 Jul 16;16:485. doi: 10.1186/s12885-016-2539-z (PMC4947293; doi:10.1186/s12885-016-2539-z)
Supplement: Additional file 10: Figure S1. — Copy number profiles of primary cultures and matched primary tumors and adjacent normal tissues. Figure S2. Morphology of VHLmut vs. VHLwt cultures. Figure S3. VHLwt cells do not express distal tubule markers. Figure S4. Xenografts derived from RCC#243mut and RCC#407mut cultures. Figure S5. Hierarchical clustering heatmap of Pearson correlation coefficients between matched VHLmut cells, VHLwt cells and primary tumor tissues. Figure S6. Functional annotation of 211 VHLmut-associated genes (red) and 382 VHLwt-associated genes. Figure S7. Enrichment map of tumor tissue vs. VHLmut culture gene expression profiles. (PPTX 10688 kb) [file 12885_2016_2539_MOESM10_ESM.pptx]

## Slide 1
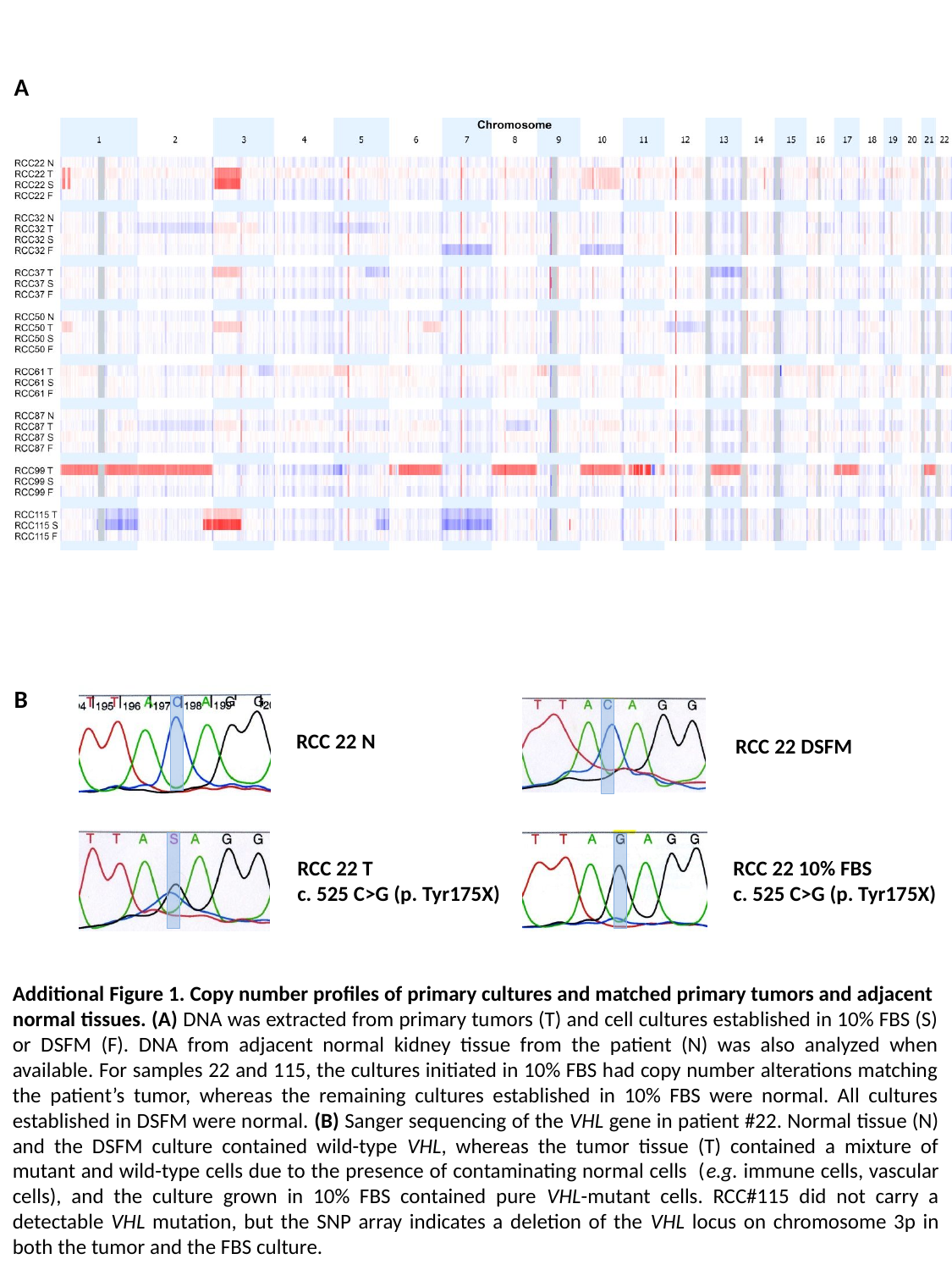

A
B
RCC 22 N
RCC 22 DSFM
RCC 22 T
c. 525 C>G (p. Tyr175X)
RCC 22 10% FBS
c. 525 C>G (p. Tyr175X)
Additional Figure 1. Copy number profiles of primary cultures and matched primary tumors and adjacent normal tissues. (A) DNA was extracted from primary tumors (T) and cell cultures established in 10% FBS (S) or DSFM (F). DNA from adjacent normal kidney tissue from the patient (N) was also analyzed when available. For samples 22 and 115, the cultures initiated in 10% FBS had copy number alterations matching the patient’s tumor, whereas the remaining cultures established in 10% FBS were normal. All cultures established in DSFM were normal. (B) Sanger sequencing of the VHL gene in patient #22. Normal tissue (N) and the DSFM culture contained wild-type VHL, whereas the tumor tissue (T) contained a mixture of mutant and wild-type cells due to the presence of contaminating normal cells (e.g. immune cells, vascular cells), and the culture grown in 10% FBS contained pure VHL-mutant cells. RCC#115 did not carry a detectable VHL mutation, but the SNP array indicates a deletion of the VHL locus on chromosome 3p in both the tumor and the FBS culture.

## Slide 2
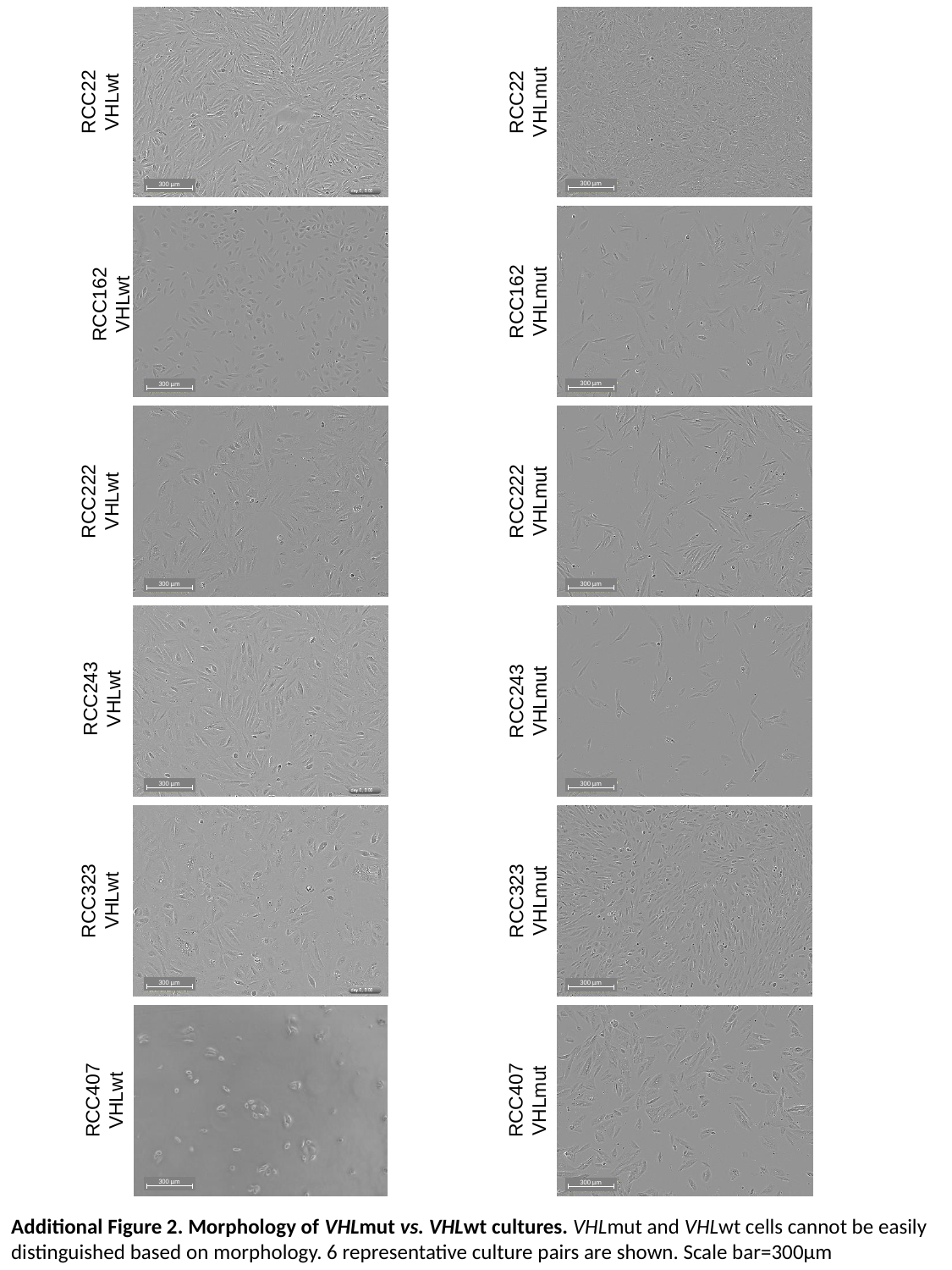

300 µm
300 µm
RCC22 VHLwt
RCC22 VHLmut
300 µm
300 µm
RCC162 VHLmut
RCC162 VHLwt
300 µm
300 µm
RCC222 VHLwt
RCC222 VHLmut
300 µm
300 µm
RCC243 VHLwt
RCC243 VHLmut
300 µm
300 µm
RCC323 VHLwt
RCC323 VHLmut
300 µm
300 µm
RCC407 VHLwt
RCC407 VHLmut
Additional Figure 2. Morphology of VHLmut vs. VHLwt cultures. VHLmut and VHLwt cells cannot be easily distinguished based on morphology. 6 representative culture pairs are shown. Scale bar=300µm

## Slide 3
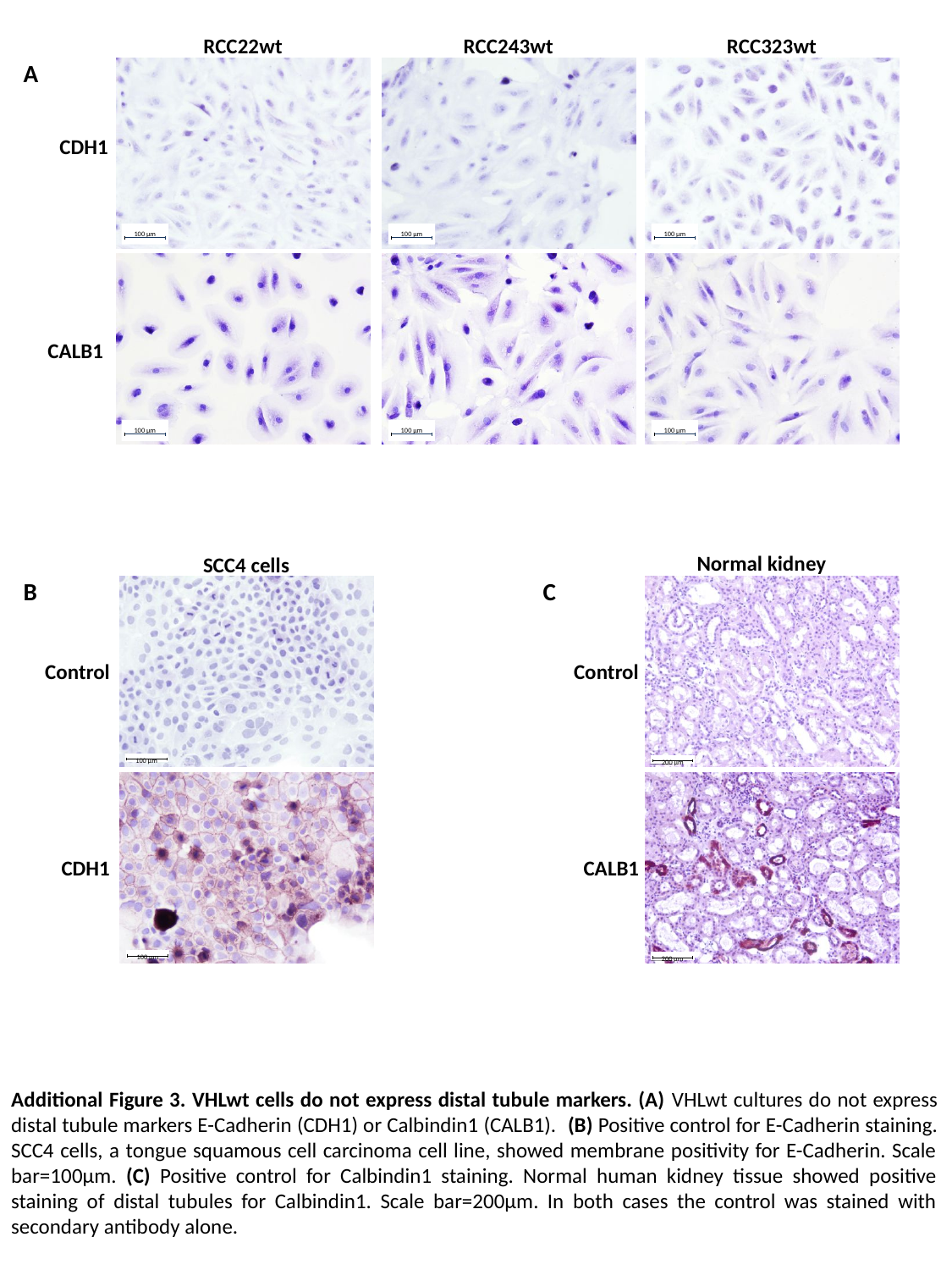

RCC22wt
RCC243wt
RCC323wt
A
100 µm
100 µm
100 µm
CDH1
100 µm
100 µm
100 µm
CALB1
Normal kidney
SCC4 cells
B
C
100 µm
200 µm
Control
Control
100 µm
200 µm
CDH1
CALB1
Additional Figure 3. VHLwt cells do not express distal tubule markers. (A) VHLwt cultures do not express distal tubule markers E-Cadherin (CDH1) or Calbindin1 (CALB1). (B) Positive control for E-Cadherin staining. SCC4 cells, a tongue squamous cell carcinoma cell line, showed membrane positivity for E-Cadherin. Scale bar=100µm. (C) Positive control for Calbindin1 staining. Normal human kidney tissue showed positive staining of distal tubules for Calbindin1. Scale bar=200µm. In both cases the control was stained with secondary antibody alone.

## Slide 4
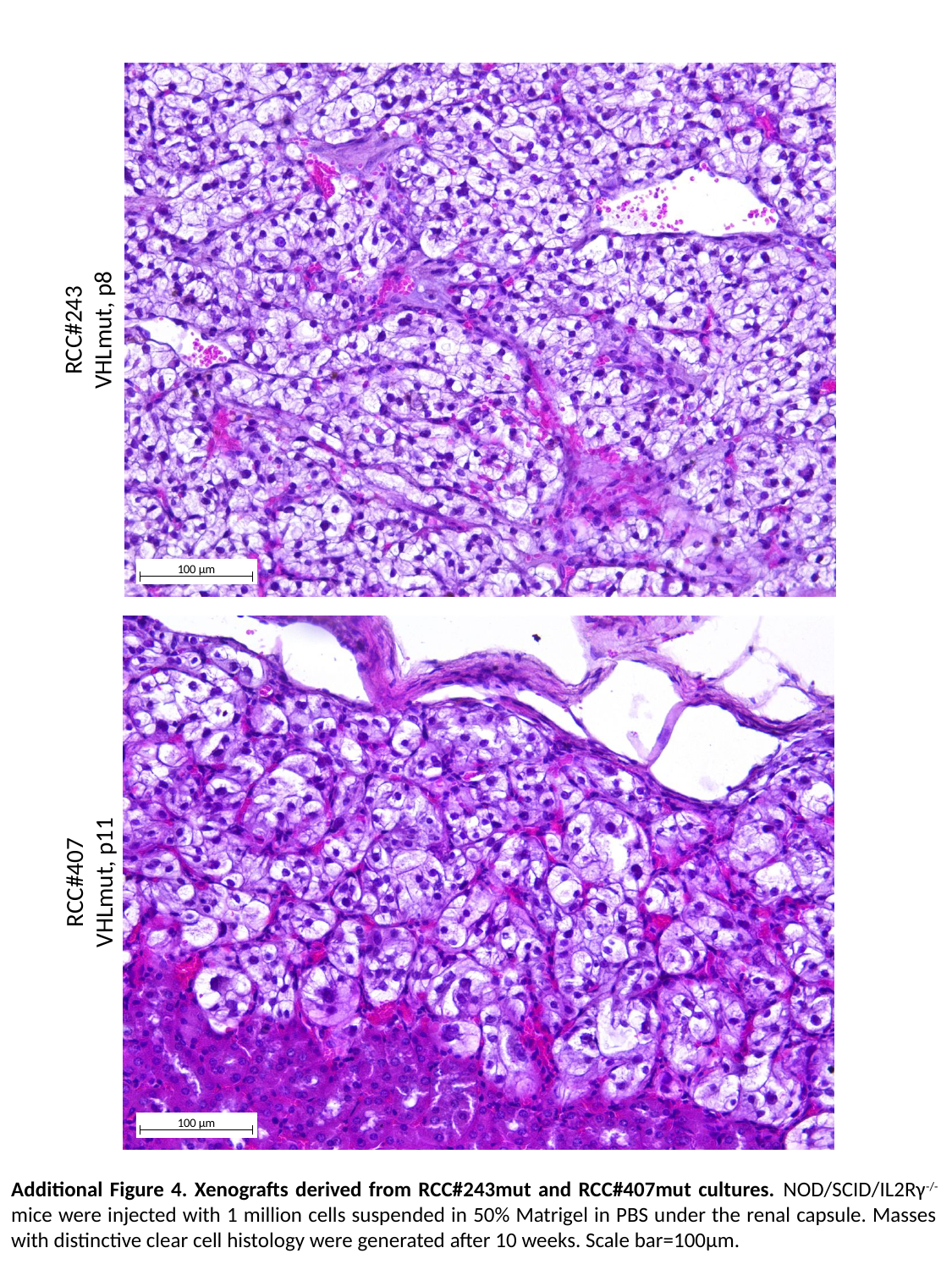

100 µm
RCC#243
VHLmut, p8
100 µm
RCC#407
VHLmut, p11
Additional Figure 4. Xenografts derived from RCC#243mut and RCC#407mut cultures. NOD/SCID/IL2Rγ-/- mice were injected with 1 million cells suspended in 50% Matrigel in PBS under the renal capsule. Masses with distinctive clear cell histology were generated after 10 weeks. Scale bar=100µm.

## Slide 5
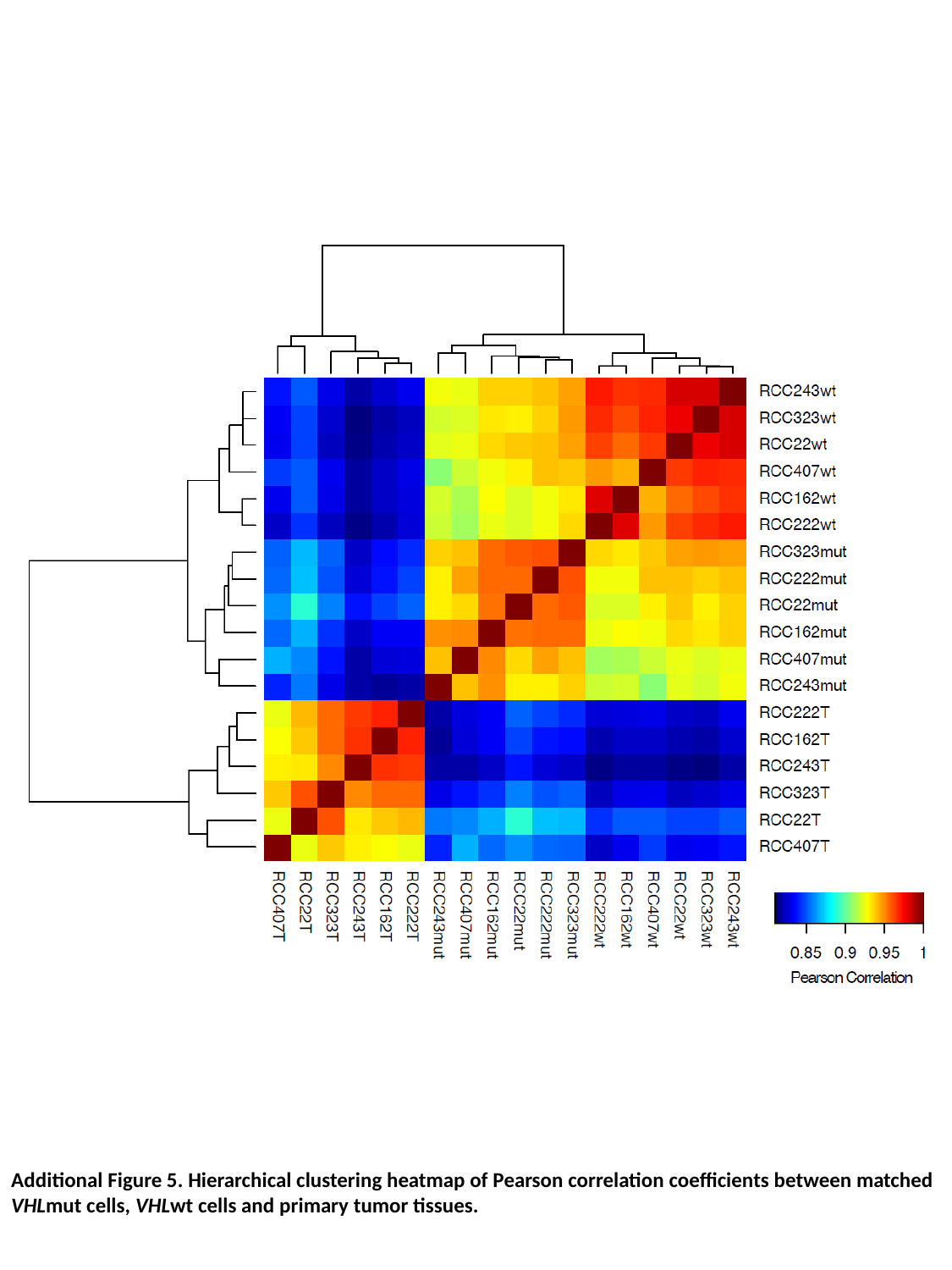

Additional Figure 5. Hierarchical clustering heatmap of Pearson correlation coefficients between matched VHLmut cells, VHLwt cells and primary tumor tissues.

## Slide 6
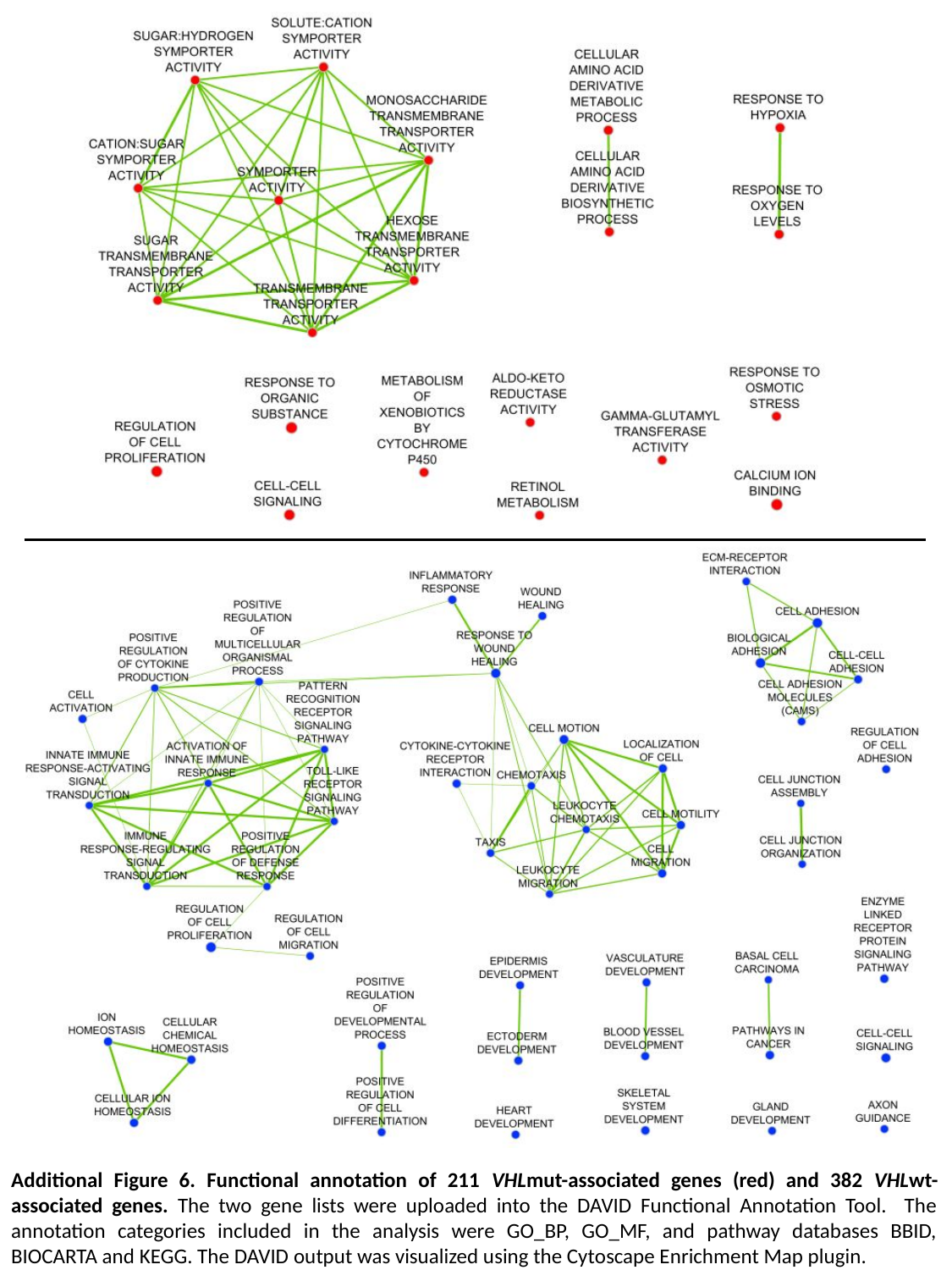

Additional Figure 6. Functional annotation of 211 VHLmut-associated genes (red) and 382 VHLwt-associated genes. The two gene lists were uploaded into the DAVID Functional Annotation Tool. The annotation categories included in the analysis were GO_BP, GO_MF, and pathway databases BBID, BIOCARTA and KEGG. The DAVID output was visualized using the Cytoscape Enrichment Map plugin.

## Slide 7
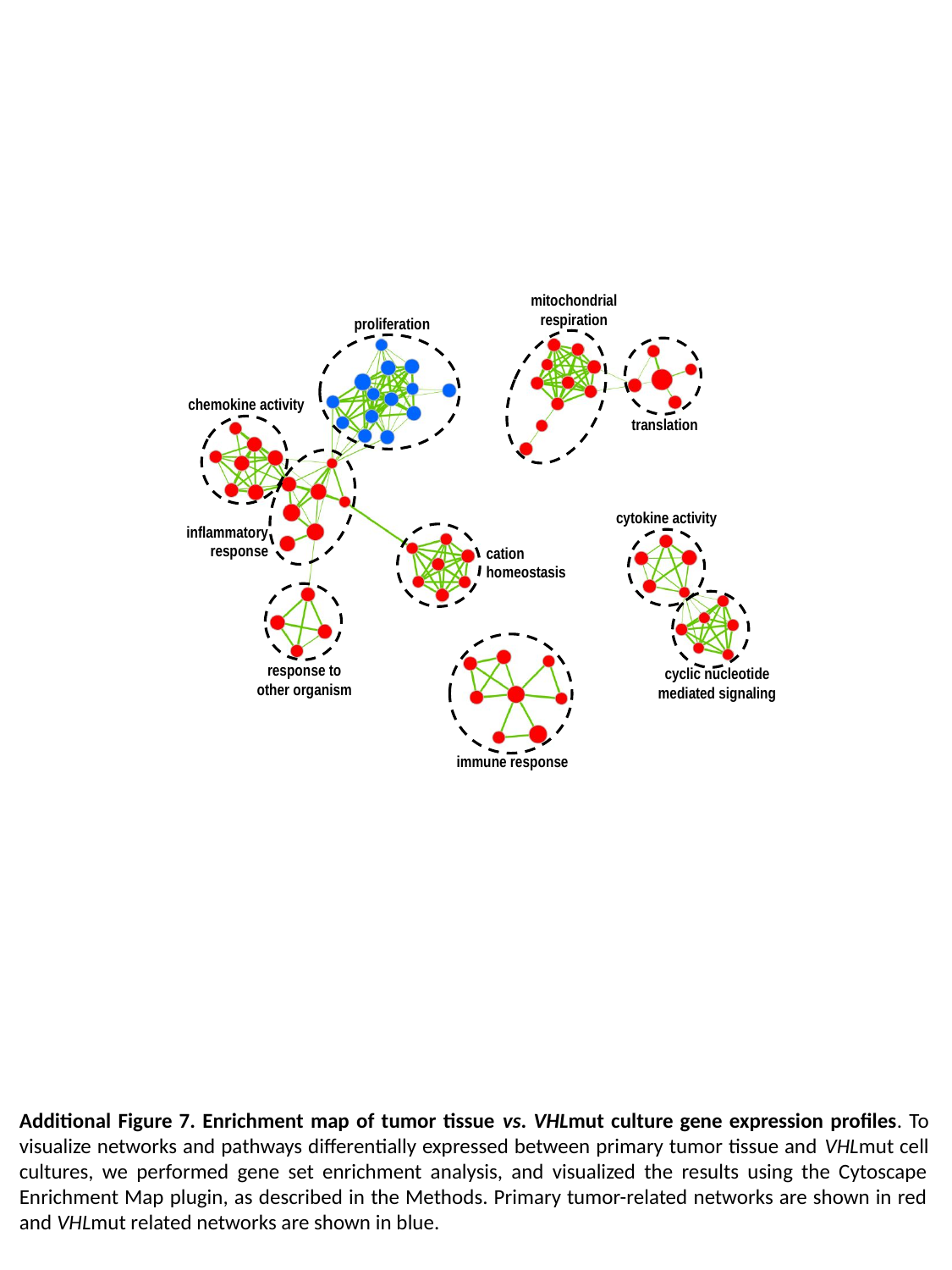

mitochondrial
respiration
proliferation
chemokine activity
translation
cytokine activity
inflammatory
response
cation
homeostasis
response to
other organism
cyclic nucleotide mediated signaling
immune response
Additional Figure 7. Enrichment map of tumor tissue vs. VHLmut culture gene expression profiles. To visualize networks and pathways differentially expressed between primary tumor tissue and VHLmut cell cultures, we performed gene set enrichment analysis, and visualized the results using the Cytoscape Enrichment Map plugin, as described in the Methods. Primary tumor-related networks are shown in red and VHLmut related networks are shown in blue.
